# Smartphone-based migraine behavioral therapy: a single-arm study with assessment of mental health predictors

**DOI:** 10.1038/s41746-019-0116-y

**Published:** 2019-06-04

**Authors:** Mia T. Minen, Samrachana Adhikari, Elizabeth K. Seng, Thomas Berk, Sarah Jinich, Scott W. Powers, Richard B. Lipton

**Affiliations:** 1 NYU Langone Health, New York, NY USA; 2 Albert Einstein College of Medicine, New York, NY USA; 3 Columbia University, New York, NY USA; 4 Cincinnati Children’s Hospital, Cincinnati, OH USA

**Keywords:** Neurology, Medical research

## Abstract

Progressive muscle relaxation (PMR) is an under-utilized Level A evidence-based treatment for migraine prevention. We studied the feasibility and acceptability of smartphone application (app)-based PMR for migraine in a neurology setting, explored whether app-based PMR might reduce headache (HA) days, and examined potential predictors of app and/or PMR use. In this single-arm pilot study, adults with ICHD3 migraine, 4+ HA days/month, a smartphone, and no prior behavioral migraine therapy in the past year were asked to complete a daily HA diary and do PMR for 20 min/day for 90 days. Outcomes were: adherence to PMR (no. and duration of audio plays) and frequency of diary use. Predictors in the models were baseline demographics, HA-specific variables, baseline PROMIS (patient-reported outcomes measurement information system) depression and anxiety scores, presence of overlapping pain conditions studied and app satisfaction scores at time of enrollment. Fifty-one patients enrolled (94% female). Mean age was 39 ± 13 years. The majority (63%) had severe migraine disability at baseline (MIDAS). PMR was played 22 ± 21 days on average. Mean/session duration was 11 ± 7 min. About half (47%) of uses were 1+ time/week and 35% of uses were 2+ times/week. There was a decline in use/week. On average, high users (PMR 2+ days/week in the first month) had 4 fewer days of reported HAs in month 2 vs. month 1, whereas low PMR users (PMR < 2 days/week in the first month) had only 2 fewer HA days in month 2. PROMIS depression score was negatively associated with the log odds of using the diary at least once (vs. no activity) in a week (OR = 0.70, 95% CI = [0.55, 0.85]) and of doing the PMR at least once in a week (OR = 0.77, 95% CI = [0.68, 0.91]). PROMIS anxiety was positively associated with using the diary at least once every week (OR = 1.33, 95% CI = [1.09, 1.73]) and with doing the PMR at least once every week (OR = 1.14 [95% CI = [1.02, 1.31]). In conclusion, about half of participants used smartphone-based PMR intervention based upon a brief, initial introduction to the app. App use was associated with reduction in HA days. Higher depression scores were negatively associated with diary and PMR use, whereas higher anxiety scores were positively associated.

## Introduction

Migraine affects over 36 million Americans and is the second most disabling condition in life adjusted years according to the World Health Organization.^1,2^ Nonpharmacologic approaches such as mind–body interventions (biofeedback and relaxation) and cognitive behavioral therapy (CBT) have Grade A evidence for migraine prevention.^3^ These therapies are effectively free of adverse effects.^3^ They have enduring benefits^4^ and may be less costly than pharmacologic interventions.^5^ However, these nonpharmacologic treatments are vastly under-utilized for a variety of factors. There are access issues: few providers are trained in these therapies for migraine, there is difficulty paying for the therapy on the part of the patients, and patients have difficulty finding the time to attend therapy appointments. A prospective cohort study of outpatients seeing a HA fellowship trained and certified headache (HA) specialist found that only about half (56.6%) of the patients initiated behavioral migraine treatment. In addition, of those who do partake in therapy, there are issues related to adherence to therapy. A recent review found few studies that attempted to assess adherence to nonpharmacologic treatments.^6^ In studies that did assess adherence, adherence was suboptimal.

To improve the uptake and initiation of behavioral therapy for HA, researchers have investigated alternative methods for delivering behavioral therapy for HA beyond the traditionally studied behavioral therapy typically consisting of 8–12 in-person sessions with a therapist. Years ago, researchers showed that prudent limited office treatment (PLOT) for the delivery of behavioral therapy, not just traditional clinic-based behavioral treatment programs, can reduce the frequency and intensity of migraine for up to 6 months.^7,8^ Long-term, PLOT is more cost effective than both traditional clinically based treatments and many preventive pharmacologic treatments.^5,9^ More recently, electronically delivered behavioral treatments for HA have been studied, however, smartphone-based electronically delivered HA interventions have not been studied to date.^10^

We sought to leverage increased smartphone technology use to integrate smartphone-based progressive muscle relaxation (PMR) for migraine. PMR was the mind–body intervention selected because it is successful as a technique that patients can do independently.^11,12^ Prior research demonstrates that pain self-management training increases self-efficacy and self-management behaviors while improving pain and depression outcomes.^13^ Prior research has also shown that most people with migraine who present for care are between ages 18 and 50 years, and between 89 and 94% of Americans in this age group have smartphones.^14,15^ Migraine patients can successfully use smartphone applications.^16^

The purpose of this study was (1) to examine whether smartphone-based, electronically delivered nonpharmacologic intervention, specifically PMR, using the RELAXaHEAD smartphone application (app) is feasible and acceptable for the self-management of migraine in a neurology outpatient setting using both quantitative methods (including back-end analytics of the app) and qualitative methods (feedback during follow-up telephone calls), (2) to explore whether smartphone-based PMR might be efficacious in reducing HA days, and (3) to examine potential predictors of app and/or PMR use to aide in future HA-related mobile health (mHealth) studies.

## Results

### Baseline data

Fifty-one patients enrolled in the study between 7 July 2017 and 12 April 2018. As shown in Table 1, 48 (94%) of the enrolled patients were female. Mean age was 39 ± 13 _SD_ years; 32 (63%) had severe migraine disability at baseline (MIDAS). Mean HA days reported per month at baseline was 13 ± 8 _SD_ (range = 4, 31), with a median of 10 HA days (IQR = 1.5) per month. All 51 (100%) patients had previously used abortive medications to stop migraine attacks and 38 (75%) had previously been on migraine preventive medication. Nine (18%) had previously been on opioids as part of their migraine treatment regimen. Fifteen (29%) of the enrolled patients had previously undergone behavioral therapy (11 CBT, 6 biofeedback, and 2 PMR) > 1 year prior to enrollment. Twenty (39%) had at least one of four overlapping pain conditions (fibromyalgia, irritable bowel syndrome, arthritis, chronic back pain). In addition, 20 (39%) reported having anxiety and 17 (33%) reported having depression. The mean PROMIS (patient-reported outcomes measurement information system) depression screen score was 50 ± 10 _SD_ (range = 31, 73) and the mean PROMIS anxiety depression score was 50 ± 10 _SD_ (range = 31, 75).

**Table 1:** Participant demographics, headache characteristics, and prior healthcare and smartphone app utilization

Participant	*N* = 51
Female	48 (94%)
Age	Mean = 39 ± 13 [19, 66]
Current (mean, SD, range)	Median 35, IQR = 21
Age when headache began (mean, SD, range)	Mean = 21 ± 12 [5, 55]
	Median=17, IQR=18
Race/ethnicity	
White/Caucasian	38 (75%)
African American	4 (8%)
Other	8 (16%)
Missing	1 (2%)
Overlapping pain conditions	
1+ pain conditions	20 (39%)
Chronic back pain	12 (23%)
Arthritis	9 (8%)
Fibromyalgia	4 (8%)
Irritable bowel syndrome	6 (12%)
Reported prior psychiatric conditions	
Anxiety	20 (39%)
Depression	17 (33%)
Positive family history of headache	39 (76%)
Headache characteristics	
Average number of headache days/month: (mean, SD, range)	Mean = 13 ± 8 [4, 31]
	Median = 10, IQR = 2
Average pain intensity (0–10 pain scale): (mean, SD, range)	Mean = 6 ± 2 [3, 10]
	Median = 6, IQR = 4
Current pain intensity (0–10 pain scale): (mean, SD, range)	Mean = 2 ± 2 [0, 7]
	Median = 2, IQR = 5
MIDAS (sum of the first five questions)	Mean = 53 ± 64 [0, 350]
	Median = 26, IQR = 2
Little or no disability (grade 1: 0–5)	0–5 = 6
Mild disability (grade II: 6–10)	6–10 = 7
Moderate disability (grade III:11–20)	11–20 = 6
Severe disability (grade IV: 21+)	21+ = 32
Psychiatric Screens	
PROMIS depression (Sum)	Mean = 50 ± 10 [31, 73]
PROMIS anxiety (Sum)	Mean = 50 ± 10 [31, 75]
Have you previously been to the emergency department for headaches?	
No visits	34 (67%)
1–5	13 (25%)
5–10	2 (4%)
>10	2 (4%)
Have you previously done any of the behavioral therapies for migraine:	
Yes	15 (29%)
Cognitive behavioral therapy	11 (21%)
Biofeedback	6 (11%)
Progressive muscle relaxation	2 (4%)
Medication usage	
Prior history of migraine preventive medications	38 (75%)
Prior history of abortive medication	51 (100%)
History opioid use	9 (18%)
History of triptan	41 (80%)
Tylenol	34 (67%)
Advil	42 (82%)
Aleve	38 (75%)
Excedrin	30 (59%)

ED = Emergency Department, App = Application

After being shown how to use the app and after doing one PMR session, participants were asked to complete the satisfaction survey. As shown in Table 2, on a Likert scale of 1–5 (where 1 = strongly disagree and 5 = strongly agree), average scores indicated that participants agreed or strongly agreed that the app was easy to use, relevant to their condition, kept their interest and attention, and that they would be happy to use it again. With regard to PMR, participants agreed or strongly agreed that they would be happy to do the relaxation again. Overall, they were neutral towards whether the relaxation helped to improve stress and low mood and whether the relaxation taught skills that will help them handle future problems.

**Table 2:** App and PMR satisfaction at baseline: number of response in each category and the corresponding Likert rating

APP and PMR satisfaction questions	Strongly disagree (1)	Disagree (2)	Neither agree nor disagree (3)	Agree (4)	Strongly agree (5)	Average Likert rating
The app was easy to use	0	0	2	27	21	4.4 ± 0.6
The information was easy to understand	0	0	26	24	0	3.5 ± 0.5
The daily diary was relevant to me to help track my headaches	0	0	11	20	19	4.2 ± 0.8
The app kept my interest and attention	0	2	4	30	14	4.1 ± 0.8
I would be happy to use the app again	0	1	2	28	19	4.3 ± 0.7
The relaxation kept my interest and attention	0	2	7	23	14	4.1 ± 0.8
The relaxation helped to improve my stress and low mood	0	3	14	18	15	3.9 ± 0.9
The relaxation taught me skills that will help me handle future problems	1	3	21	17	8	3.6 ± 0.9
I would be happy to do the relaxation again	0	1	1	24	24	4.4 ± 0.6

### Feasibility acceptability results

The quantitative HA diary data and PMR use data were obtained from the RELAXaHEAD application. Tables 3 and 4 show composite diary and PMR usage. Diary was used on average 46 ± 33 _SD_ (range = 1, 90) days by each participant. Of the 1053 total uses of the diary by all participants, 665 (63%) days were reported as HA-free days. Over the period of 90 days 63% (370/612) of per week total diary uses among the 51 users were 1+ time/week and 58% (338/612) of uses were 2+ times/week. PMR was played on average 22 ± 21 _SD_ (range = 1, 76) days by each participant, with a mean per session duration of 11 ± 7 _SD_ min. The average number of days of the PMR short and long audio files played were similar: 14 ± 16 _SD_ versus 14 ± 15 _SD_, respectively. Over the period of 90 days, 47 percent (260/612) of total PMR uses per week (among 51 participants) were 1+ time/week and 35% (179/612) of uses were 2+ times/week.

**Table 3:** PMR composite data

Electronic PMR data entered	
Total no. days of PMR played (per participant)	Mean = 22 21 [1, 76]
	Median = 12, IQR = 24
No. of days of PMR played short file	Mean = 14 ± 16 days/person [1, 56]
	Median = 7, IQR = 18
No. of days of PMR played long file	Mean = 14 ± 15 days/person [1, 49]
	Median = 9, IQR = 16.5
Total time PMR played/day (in mins)	Mean = 10 ± 7 [0.02–22]
	Median = 8 mins, IQR = 11

**Table 4:** PMR and diary composite data for completer analysis (excluding dropouts)

Electronic PMR data entered per participant	
Total no. days of PMR played (days/participant)	Mean = 23 ± 22 [1, 76]
	Median = 12, IQR = 35
No. of days of PMR played short file	Mean = 16 ± 17 days/person [1, 56]
	Median = 8, IQR = 23
No. of days of PMR played long file	Mean = 16 ± 16 days/person [1, 49]
	Median = 9, IQR = 23
Total time PMR played/day (in mins)	Mean = 11 ± 7 [0.02–22]
	Median = 8, IQR = 11
Total no. days diary entered per participant	Mean = 51 + 33 [1, 90]
	Median = 56, IQR = 66
Headache-free days entered (overall)	727

**Fig. 1:**
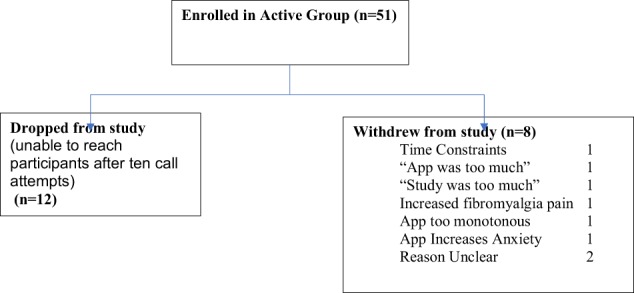
NYU-migraine participants enrolled from 7 July 2017 to 12 April 2018

**Fig. 2:**
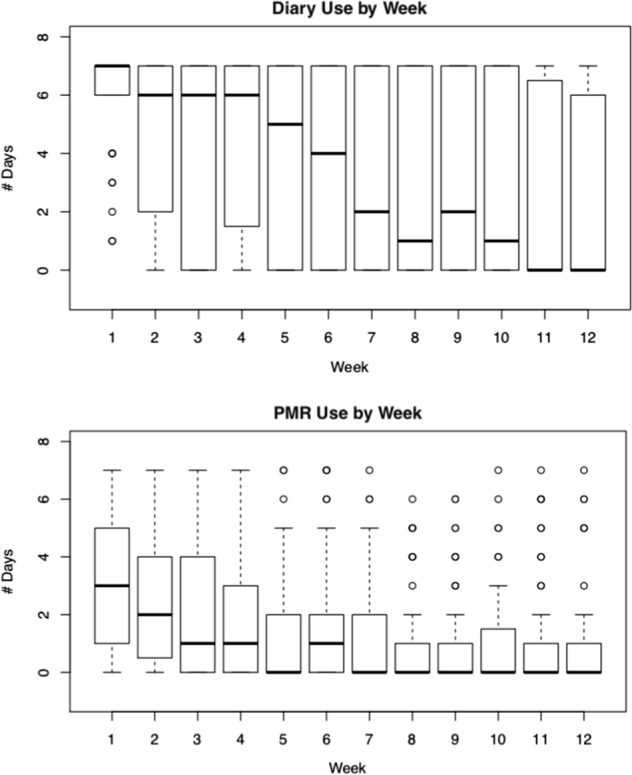
Weekly diary and PMR use. Boxplots of distribution of number of days the diary was used (top figure) and the PMR was used (bottom figure), every week for participants enrolled in the study. For each boxplot, the solid dark center line represents the median, the bounds of the box are given by the first quartile (Q1) and the third quartile (Q3), and the whiskers are bounded by (Q1 − 1.5 × IQR, Q3 + 1.5 × IQR). The points outside the whiskers are the outliers. The median number of days used per week has a decreasing trend over weeks since enrollment, for both diary use and PMR use

As shown in Fig. 1, nine participants withdrew from the study before the completion of the 90-day period. As shown in Tables 5 and 6 and Fig. 2, all 51 (100%) participants used the diary in the first week, however, the usage declined to 32 participants (63%) in week 6 and to 25 participants (49%) in week 12. Forty-three participants (84%) used the PMR in the first week, and 26 (51%) used it in week 6 and 15 (29%) used the app in week 12. Usage declined by week; most users of the app/PMR used it in the first 6 weeks. Completer analysis was performed to study usage of PMR and diary, excluding the nine participants who withdrew from the study. Small changes are observed in the average days used per participant for both PMR and diary. Mean days per participant increased by one day for overall PMR use, whereas that for diary use increased by 6 days.

**Table 5:** Count and percentage of users with non-zero use per week after enrollment

	PMR users, *n* (%)	Diary users, *n* (%)
Week 1	43 (84%)	51 (100%)
Week 2	38 (75%)	45 (100%)
Week 3	30 (59%)	35 (69%)
Week 4	30 (59%)	39 (76%)
Week 5	23 (54%)	34 (67%)
Week 6	26 (51%)	32 (63%)
Week 7	21 (41%)	29 (57%)
Week 8	21 (41%)	28 (55%)
Week 9	18 (35%)	30 (59%)
Week 10	16 (31%)	26 (51%)
Week 11	16 (31%)	25 (49%)
Week 12	15 (29%)	25 (49%)

**Table 6:** Distribution of PMR days per week and diary days per week among 51 participants for 90-day period

PMR days/week	0	1	2	3	4	5	6	7
Count	352	81	59	44	36	43	27	21
Percentage	53%	12%	9%	7%	5%	7%	4%	3%

Diary days/week	0	1	2	3	4	5	6	7
Count	242	32	31	23	27	22	44	242
Percentage	37%	5%	5%	3%	4%	3%	7%	37%

The qualitative data were obtained during the follow-up telephone calls. Table 7 and Box 1 show the data collected during the follow-up calls to participants. During the follow-up calls, obstacles to participating in the smartphone-based PMR intervention were recorded. Key themes included: Disliking the PMR Behavior/Audio File, Difficulty Maintaining the PMR Practice, Technical Issues. In addition, while responding regarding potential obstacles, participants sometimes reported Positive Opinions of PMR, which served as an additional theme.

**Table 7:** Follow-up phone call data: neuro-migraine active participants (*n* = 51)−withdrawals (*n* = 8) = participants analyzed (*n* = 43)

Reported doing the relaxation therapy	1-month follow-up	2-month follow-up	3-month follow-up
Yes	25	19	13
No	5	2	6
Uncertain (unanswered)	13	22	24
Representative comments about doing the relaxation therapy	• Prefers to do it by herself, without audio file. Too repetitive. Hire voice actor to do audio file, voice not too relaxing. • Thinks it is great but it does not make any difference in her symptoms. Does not do it as much as she should. • Enjoys it a lot, just hard to get 20 min in because she is busy. Helps her concentrate on relaxing her muscles because she did not realize she was tensing them so much. Found it complements the botox, found it incredibly helpful (started both the same day). Principles of relaxation in general help her relax muscles in neck or face. • Does the relaxation from memory—does arm, neck, head exercises. Parts of it are helpful doing 20 min a day “drove me nuts” but helpful for tension. • All for it. Started again today after vacation. Been very helpful. Generally felt better in terms of pain and stress. • Does not like it. Finds the app very frustrating and difficult to use. Do not think therapy is helping with headaches but would not mind using it just in case. • Thinks it is useful, would be nice if it had variations in it. It would be nice to have something else to listen [to]. • Only likes short one. Long one too long for her to relax • Thinks it is helpful and also helpful to track headaches. • Hard because very repetitive. Not been able to listen every day to both. Frustrated by repetitiveness. Had a great month. • Really enjoys it. Helps with managing other types of stress. Going through PMR made accustomed, and now is part of day to day activities. Starts and ends day well. Helps get “better quality of life”. If feeling overwhelmed will do PMR. • Longer session works better. Still get headaches but not as severe. Would like to see more of neck and back relaxation. • Like it! Helped with breathing. Helped with relaxation. • To be honest it is getting a little boring, doing the same thing over and over again. Do the short one somewhat regularly.		
Recommend the therapy	1-month follow-up	2-month follow-up	3-month follow-up
Yes	19	15	13
No	1	1	0
Uncertain	5	2	0
Unanswered	18	25	30
Representative comments about recommending the therapy	• Can not honestly tell if it is affecting the frequency of migraine but can recommend. • Has not seen a difference yet. Generally, relaxation is a good thing to do once a day. • No, because of the set[-up] of the app. Not the technique. • Not necessary but if they fixed the [app] then maybe. Right now, it is very frustrating. • Would recommend for people who do not want to try traditional methods, like medication botox etc. Would not be first thing she recommends though. • Yes, felt like it helped. • Yes, if people get into a good routine it could be very helpful. • Would recommend trying at least to see. • Thinks it is helping, if she starts to get a headache she tries to do 3 min and she feels that it does reduce the intensity. • For her, it has not made any difference in her symptoms. • Yes, for a meditative aspect.		

### Initial efficacy assessments

**Fig. 3:**
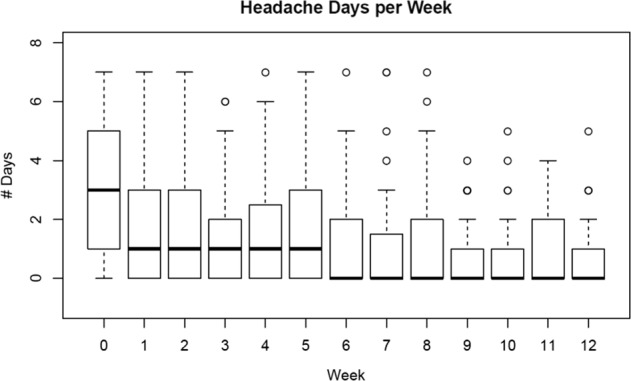
Headache days reported per week. Boxplots of distribution of headache days reported each week since enrollment for participants who used the diary in the corresponding week. Week 0 is the week of enrollment. For each boxplot, the solid dark center line represents the median, the bounds of the box are given by the first quartile (Q1) and the third quartile (Q3), and the whiskers are bounded by (Q1 − 1.5 × IQR, Q3 + 1.5 × IQR). The points outside the whiskers are the outliers

**Fig. 4:**
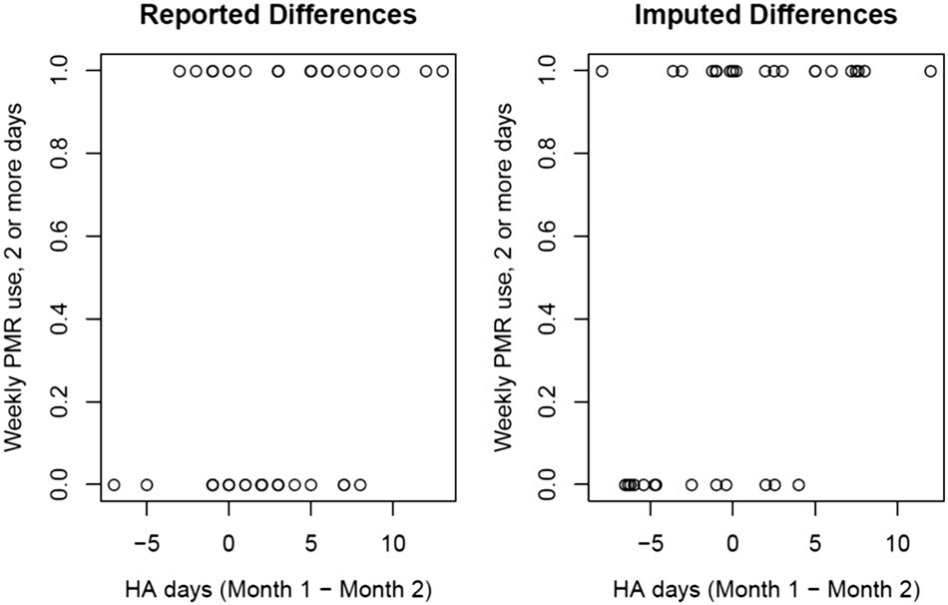
Difference in headache days between months one and two in high and low users. The first display in the left shows the observed difference in headache days reported by each user, whereas that in the right shows the imputed difference. Users are categorized as high users if their weekly PMR use is two or more days, and as low users otherwise. Along the *x* axis is the difference in headache days, and along the *y* axis is whether the weekly PMR use is greater than or equal to 2 days

Figure 3 shows the plot of average number of reported HA days by week. On average, fewer HA days were reported over time (though this could be that participants were not using the diary or the app as often in later weeks as demonstrated in Fig. 2). Figure 4 shows the plot of difference in HA days for high (*y* axis = 1) and low (*y* axis = 0) PMR users. On average, high users had 4 fewer days of reported HAs in month 2 compared with month 1, whereas low PMR users had only 2 fewer HA days in month 2. The difference in changes in HA days for low and high PMR users was significantly different than zero (*p* value = 0.02). With the informative missingness, using the same approach as above to compare changes in HA days in high PMR users and low PMR users, the mean HA days were reduced by 2 days in high PMR users from month 1 to 2, however, mean HA days increased by 3 days in low PMR users. Reduction in HA days from month 1 to 2 for high PMR users was different than for low PMR users (*p* value = 0.002).

### Prediction modeling

PROMIS depression score, PROMIS anxiety score and response to a questionnaire about whether the app maintained their interest remained in the prediction model post selection for both PMR outcome and Diary use outcomes. PROMIS depression score was negatively associated with the log odds of using the Diary at least once (versus no activity) in a week (odds ratio = 0.70, 95% CI = [0.55,0.85]). PROMIS anxiety (odds ratio = 1.33, 95% CI = [1.09, 1.73]) and the satisfaction questionnaire about the app keeping interest (odds ratio = 7.76, 95% CI = [1.66,46.42]) were both positively associated with using the Diary at least once every week. Similar results were observed for PMR use, with odds ratios of 0.77 (95% CI = [0.68, 0.91]) for PROMIS depression score, 1.14 (95% CI = [1.02, 1.31]) for PROMIS anxiety and 5.4 (95% CI = [1.22, 20.90]) for app keeping interest questionnaire, respectively.

Area under the curve (AUC) for the diary use model was 0.98, whereas that for the PMR use model was 0.96, both indicating a good prediction performance of the model.

Box 1 Themes and subthemes for when participants were queried about what obstacles they encountered when performing the smartphone-based PMR **Disliking the PMR behavior/audio files**

App-specific complaints

- Redundancy of PMR (5)

- Dislike of audio/voice recording (3)

PMR as a therapy

- Inability to sit still/lack of focus for long PMR (5)

- Able to do relaxation without audio (2)

Physical effects of therapy

- Increases tension (1)

- Painful to use during headache or migraine (2)

- Increases tiredness (1)

- Visual disturbances (1)

- Increases anxiety (1)

- Uncomfortable with breathing exercises (1)

**Difficulty maintaining the PMR practice**

Time/logistical constraints

- Time management (17)

- Difficult to schedule both PMR sessions (6)

- Unable to find time for long PMR (5)

- Difficult to find private space to complete PMR (4)

- Vacation (2)

- Other health problems (3)

Behavioral obstacles

- Lack of self discipline (2)

- Forgets to complete PMR (5)

**Technical issues**

- Personal phone issues (2)

- Technical issues with app (1)

**Positive opinions on PMR**

- Enjoys short PMR (4)

- Enjoys long PMR (1)

- Enjoys PMR prior to bedtime (3)

## Discussion

In the first study exploring smartphone-based PMR on neurology patients with migraine, we found that participants who meet criteria for migraine preventive therapy were willing to try a low cost, traditionally evidence-based minimal intervention behavioral therapy delivered via a smartphone application. We had the following key findings: (1) About one out of every two participants demonstrated uptake of the smartphone-based PMR intervention based upon a brief, initial introduction to the app. (2) Participants averaged ~11 min of PMR on the days that they used it. (3) There appeared time limited acceptability of the intervention by 6 weeks. (4) There appeared to be a dose relationship in using PMR and a decrease in HA days. Personal characteristics, however, which were not examined in this study, may be associated with adherence and may confound the study.

Given that this was a low-cost study with an uptake rate of one out of two, we believe this initial study demonstrates feasibility, especially given the fact that so many people do not receive psychological treatments for pain owing to barriers to access such as a shortage of providers, expense, and geographic distance from treatment centers.^17^ We showed that in a population of migraine with the majority having severe migraine disability, 84% used the PMR in the first week and 51% used in at week 6. This is significantly better than a prior study of 221 severe tertiary care HA inpatients who were advised to do relaxation therapy in the 7-day period before discharge, in which only slightly more than the majority (59%) used relaxation as a preventive measure.^6^

Participants averaged 11 min of PMR use on the days in which PMR was used. There is no known dose that is considered most efficacious across migraine behavioral treatment trials, let alone behavioral treatment studies in general. Although Jacobson initially developed PMR to be used for an hour each day, it was later modified to be used for briefer periods of time.^18^ We trialed the PMR dose duration in this population based on the SMILE study.^19,20^ Dosing decisions must be made based on feasibility.^21^ The fact that people were willing to do the PMR for a shorter duration is not surprising. We did not ask patients to do the PMR the way it is typically taught in clinical practice; we did not use the gradually shortened PMR, which reduces 16 muscle groups down to 4 muscle groups time nor did we use PMR by recall.^22^

There was an initial signal that there may be a dose–response relationship between PMR use and a decrease in HA days. Many participants were not reporting HA days in month 2 compared with 1 in low PMR user group and our imputation accounted for that. We believe that the increase in HA days for low users from month 1 to 2 using imputation method is the consequence of underreporting in month 2 stipulation.

Overall, the usage is similar to rates of adherence in other migraine-preventive studies, including both preventive behavioral and migraine medication studies. The discontinuation rate for migraine medication preventive treatment is >44% based on claims database studies.^23^ Reasons for discontinuation of internet-based psychotherapies for pain previously cited in the literature include being disenchanted or seeking a better alternative,^24^ health problems and illness, difficulty using a computer or being physically uncomfortable using a computer, and personal problems.^25^ This is similar to our reasons for withdrawal which were: disenchanted, time, and worsened symptoms. In addition, our withdrawal rate of 16% (eight participants formally withdrew from the study), is similar to rates of attrition of other internet-based studies of psychological interventions for pain. A Cochrane review of internet-based psychologic therapies for pain showed that the overall attrition was 17.4% on average (range 0–25).^25^ The studies in the Cochrane review had a mean duration of 11 weeks (range 3–46 weeks). This is a much lower rate than the dropout rate of a third of recruited patients in traditional randomized controlled trials (RCTs) of pharmacological treatments, with rates even > 80% in some long-term relapse prevention trials.^24^

By 6 weeks, it was clear that user engagement diminishes over time. This is in line with other mHealth studies, which tend to have short lengths. Ninety days may not be feasible for patients nor realistic or clinically applicable per expert opinion from HA behavioral specialists who state that by four weeks, participants would be able to learn the PMR technique and integrate it into their lives. Adjustments may be made to the protocol so that the study duration might be 6 weeks and participants may be asked to participate for fewer days of the week.

In our study, depression was a negative predictor for use of the app and PMR, whereas anxiety was a positive predictor of use. This is not surprising given that a prior study showed that those with lower levels of depression did better in a RCT using an online CBT intervention.^26^ Outcomes can be tied to motivation, symptom severity, and patient expectancy.^26^

To ensure the best quality data and to maximize chances of proving efficacy, many study designs seek motivated participants. They screen outpatients after a certain baseline period. They may also screen out those who do not show for initial enrollment sessions and thus do not re-schedule prospective participants. They may also leave it to the personal interview or interviewer to judge that a person would not be a good candidate for a behavioral trial. In order to be as pragmatic as possible, we did not do any of the above—we included all of those who demonstrated some willingness to engage in research. In addition, most studies do not include those who are as severely disabled as our study population and/or do not include people who have failed as many preventive treatment regimens.

In the prior Cochrane review of internet-based psychotherapy for pain, for those who remained in the studies, overall compliance rates with interventions, e.g., number of sessions completed are generally not reported and planned analyses of secondary outcomes (quality of life and acceptability/satisfaction) were limited because data were limited.^25^ We had back-end analytics to capture what other studies have not had the capability to do.

The study had several limitations. This was a pilot study with 51 subjects, and it was a single-arm feasibility study not powered to examine efficacy. Although a strength of the study was that we had back-end analytics to control for the amount of time playing the PMR, it was not possible to ensure that participants were fully engaged and as prior researchers note in their studies, it is not feasible to control for multitasking.^27^ In addition, we did not control for changes in meds during the study period.

This was a rigorous study design that focused exclusively on smartphone-based PMR and may not reflect what expert clinicians use in actual clinical practice. In the case of PMR, some HA behavioral therapists will start with the full PMR (up to 16 muscle groups) and will then gradually lessen the number of muscle groups over a brief period of time (approximately two weeks). Others may start with a short version and have people gradually increase the duration of PMR over time. They will also move from practiced PMR with audio-recordings to the PMR being done by self-recall. This is an important area to investigate both because participants may be dissatisfied with the intervention (real “dropouts”) or because the intervention has met their needs (attainers).^24^ Larger funded studies may compare such factors. Also, much of the current research and clinical practice for migraine behavioral intervention involves multiple components. Future work might also investigate the use of additional behavioral techniques such as deep breathing or imagery in addition to the PMR.

We might also test additional strategies for engaging participants in the therapy. We had difficulty reaching participants during the follow-up calls. Almost three quarters of enrollment (37/51) took place for the study before funding was received to hire a part-time paid research coordinator. Prior research has found that migraine patients may accept up to five prompts per day for electronic diary keeping. Beep reminders with time limits on the silence mode to a maximum of 2 h and voice prompts might be used.^28^ In addition, gamification may also help to improve adherence to the PMR. Future studies may examine how to improve/better engage those with higher depression scores or may trial other methods for these patients. Future studies may also seek to target those with migraine and comorbid anxiety to optimize treatment efficacy.

In conclusion, mHealth studies are at an advantage of some other behavioral studies in being able to monitor the usage patterns by tracking the frequency, duration, and length of the intervention. In addition, mHealth interventions have high fidelity: the same intervention is offered to all the participants.^24^ We found that in a convenience sample of 51 patients, more than half of whom had severe migraine, about one of every two patients demonstrated engagement with smartphone-based PMR intervention based upon a brief, initial introduction to the app. Also, there appeared to be time- limited acceptability of the intervention by 6 weeks. This is a promising area given its low cost, scalable method, and future studies can begin to examine efficacy.

## Methods

### Study design

Feasibility and acceptability of the intervention and predictors of the RELAXaHEAD app diary use and performance of PMR were examined in a single-arm prospective study of 3 months duration, with baseline, 1-, 2-, and 3-month measurements.

The study was approved by the NYU Medical Center Institutional Review Board, and written informed consent was provided by research volunteers.

### Recruitment

From 7 July 2017 to 12 April 2018, participants were recruited from the New York University Langone Health Neurology practice. The neurology setting was selected as nearly a quarter (23.2%) of people with migraine who present for care present to specialty outpatient settings, presumably neurology or HA specialty clinics.^29^ NYU-approved research volunteers pre-screened prospective subjects using Epic^30^ and then approached patients and/or their physicians to inform them that the patient may be eligible for a study examining electronic treatment for migraine prevention. Participants were recruited using convenience sampling. In order to be eligible, patients had to be between the ages of 18–80 years, speak English, own a smartphone, have a diagnosis of migraine from a neurologist (and confirmed using International Classification Headache Disorders criteria via the comprehensive questionnaire^31^), self-report four or more HA days/month, be willing to engage in a smartphone-based behavioral therapy, and be free of behavioral therapy (CBT, biofeedback or PMR) for migraine in the past year. If eligible, research volunteers provided signed informed consent.

### Intervention

PMR, a standardized, evidence-based behavioral treatment used for migraine since the 1980s^32,33^ was selected as the behavioral intervention because of its success as a technique that patients can do independently.^11,12^ The RELAXaHEAD app was modeled after the app used in the SMILE study that examined smartphone-based PMR for epilepsy patients.^19,20^ (It has the same PMR used in that study and the Human Epilepsy Project (HEP) app.)^34^ There is an ~ 5-minute PMR session and an ~ 15-minute PMR session embedded in the app. For the RELAXaHEAD app, modifications were based on input from migraine patients and HA specialists using methods described elsewhere.^35^ Our RELAXaHEAD app has back-end analytics built in to record the amount of time spent playing the PMR. Pain intensity, duration, and medications taken may be recorded using the app. In addition, the RELAXaHEAD app allows the use of reminders and timely follow-up of non-compliant participants via real-time investigator data monitoring capabilities. The RELAXaHEAD application uses a platform developed by IRODY, and NYUMC MCIT previously approved its development. Participants were asked to use the app for 90 days by completing the HA diary daily and performing PMR using the app for 20 min/day. To cover data plan charges associated with use of their personal phones, participants who enrolled after the funding date (1 November 2017) were paid $25 at enrollment and $1/day for each day that data were entered into the app.

### Measurements

Primary outcome measures included the following quantitative data: (1) number of days of HA diary use/90-day period, (2) number of days of PMR use/90-day period, and (3) dose (minutes) of PMR use/day in those who used it. These data were abstracted from back-end report maintained by the app.

Secondary outcomes were (1) the number of HA days recorded in the app for the high and low PMR users and (2) predictors of app use and PMR use. App diary (or PMR) use for every week following the enrollment was determined based on whether the participant used the diary (or the PMR) at least once a week in a given week during the 90-day period.

Predictors of app diary use and performance of PMR included age, gender, race, self-reported age at first HA, self-reported number of HA days in the past month, self-reported average HA intensity, self-reported current HA intensity at time of enrollment into the study, total MIDAS based on the five-question score, self-reported usage of behavioral therapy for migraine prior to 1 year of enrollment in the study (yes/no), PROMIS depression, PROMIS anxiety, self-reported presence of the overlapping pain conditions studied (yes/no), self-reported use of any migraine preventive medicines (yes/no), the response (in Likert scale) to the satisfaction questionnaires asked during the enrollment, and whether they were compensated for participation in the study.

Specific measures were as follows:

The MIDAS^36^ is a validated five-item questionnaire that has internal consistency and test–retest reliability and was developed to assess HA-related disability with the goal of improving migraine care. Questions ask about prior activity limitations over the past 3 months. Examples include “On how many days in the last 3 months did you miss family, social or leisure activities because of your HAs?” and “On how many days in the last 3 months did you miss work or school because of your HAs?”

The PROMIS depression and anxiety questionnaires are disease agnostic questionnaires developed by the National Institutes of Health to assess depression and anxiety, respectively, over the prior seven days.^37,38^ They are calibrated by each item question. The PROMIS depression assesses self-reported negative mood, views of self, social cognition, and decreased positive affect and engagement. Somatic symptoms are not included so as not to be confused with potential confounders, e.g., changes in sleep. The PROMIS anxiety measures self-reported fear, anxious misery, hyperarousal, and somatic symptoms related to arousal. Behavioral avoidance is not emphasized.

The satisfaction questions were developed specifically for this study by the HA specialist and appear in Table 2.

Data were collected by the research study team at baseline (in-person enrollment), from the app, and during 1-, 2-, and 3-month follow-up telephone calls. We also collected qualitative data during these phone calls. During the follow-up telephone calls, participants were asked the following three open-ended questions: 1. What do you think of the relaxation therapy? 2. What obstacles have you encountered in doing the therapy as recommended? 3. Would you recommend the therapy to others with migraine?

### Analysis

Descriptive statistics were used to describe baseline participant characteristics and use of the app diary and PMR. Use of the app was described in terms of the mean number of days the diary was used in the 90-day period. PMR performance was described in terms of the mean number of days PMR was used, mean duration of PMR, and proportion of short (~5-minute long PMR) and long (~15-minute PMR) PMR sessions during the 90-day period.

Logistic regression was used to explore predictors of app diary and PMR performance. Two stage mixed effects logistic regression model, with random effects for each participant and for each study week, was used to account for repeated measures per participant and potential differences across study weeks since enrollment. The outcome variable was a dichotomous variable of whether the participant used the diary (or the PMR for predicting PMR use) at least once in a given week during the 90-day period. In the first stage, the best set of predictors were selected using Likelihood-ratio tests with a backward selection procedure to avoid overfitting. There were estimates of odds ratio of the fixed effects using the parsimonious set of predictors post selection in the second stage of the logistic regression. AUC measure was computed using the final model to assess how well the fitted model predicts PMR and diary use.

We also sought to examine when the diary and PMR were most likely to be used over the 90-day period. This was assessed by summing the number of days/week of diary and PMR use.

We assessed whether there was a signal of efficacy of PMR use in the outcome of HA days by comparing the changes in total number of HA days reported on the diary from month 1 to month 2 for high and low PMR users in month 1, using a two-sided *t* test. (High PMR users were any user with two or more weekly uses in the first 4 weeks of enrollment.) The first analysis of efficacy assumed that the days in which the diary was not used are HA-free days. However, to account for informative missingness, we also imputed HA days in the second set of analysis. For participants with at least one reported HA day in a month post enrollment, we computed rate of reported HA days as a ratio of observed number of HA days and total number of days the diary was used. Expected HA days per month was then computed as a product of the rate of HA days and the total number of days in a month for each participant, and for months 1 and 2, respectively. Sixteen participants did not use the diary at all in month 2. These participants were excluded from the analysis that used imputed HA days.

Using the data collected during the follow-up calls, qualitative analyses were conducted using general thematic analysis, allowing themes and subthemes to emerge iteratively. Contextual evaluation was accomplished as two members of the study team individually created a list of codes for each patients’ response to the obstacles question and then collaboratively compared and agreed upon codes with the help of a HA expert with experience in qualitative research. Based upon the codes, two study team members then developed themes and subthemes with the help of the same HA expert with qualitative research experience.

### Reporting summary

Further information on research design is available in the Nature Research Reporting Summary linked to this article.

## Supplementary information

Reporting Summary

## Data Availability

Upon request, data supporting the findings of this study are available from the corresponding author.

## Supplementary information

**Supplementary information** accompanies the paper on the *npj Digital Medicine* website (10.1038/s41746-019-0116-y).
